# Effect of teriparatide on drug treatment of tuberculous spondylitis: an experimental study

**DOI:** 10.1038/s41598-022-25174-6

**Published:** 2022-12-15

**Authors:** Subum Lee, Ye-Jin Seo, Je-Yong Choi, Xiangguo Che, Hyun-Ju Kim, Seok-Yong Eum, Min-Sun Hong, Sun-Kyoung Lee, Dae-Chul Cho

**Affiliations:** 1 Department of Neurosurgery, Korea University Anam Hospital, Korea University College of Medicine, Seoul, Republic of Korea; 2 Department of Neurosurgery, School of Medicine, Kyungpook National University, Kyungpook National University Hospital, 130 Dongduk-Ro, Jung-Gu, Daegu, Republic of Korea; 3 Department of Biochemistry and Cell Biology, School of Medicine, Kyungpook National University, Daegu, Republic of Korea; 4 Division of Immunopathology and Cellular Immunology, International Tuberculosis Research Center, Gyeongsangnam-Do, Changwon-Si, Republic of Korea

**Keywords:** Drug development, Experimental models of disease, Preclinical research, Translational research, Infectious diseases, Musculoskeletal system

## Abstract

Tuberculous spondylitis often develops catastrophic bone destruction with uncontrolled inflammation. Because anti-tuberculous drugs do not have a role in bone formation, a combination drug therapy with a bone anabolic agent could help in fracture prevention and promote bone reconstruction. This study aimed to investigate the influence of teriparatide on the effect of anti-tuberculous drugs in tuberculous spondylitis treatment. We used the virulent *Mycobacterium tuberculosis* (Mtb) H37Rv strain. First, we investigated the interaction between teriparatide and anti-tuberculosis drugs (isoniazid and rifampin) by measuring the minimal inhibitory concentration (MIC) against H37Rv. Second, we evaluated the therapeutic effect of anti-tuberculosis drugs and teriparatide on our previously developed in vitro tuberculous spondylitis model of an Mtb-infected MG-63 osteoblastic cell line using acid-fast bacilli staining and colony-forming unit counts. Selected chemokines (interleukin [IL]-8, interferon γ-induced protein 10 kDa [IP-10], monocyte chemoattractant protein [MCP]-1, and regulated upon activation, normal T cell expressed and presumably secreted [RANTES]) and osteoblast proliferation (alkaline phosphatase [ALP] and alizarin red S [ARS] staining) were measured. Teriparatide did not affect the MIC of isoniazid and rifampin. In the Mtb-infected MG-63 spondylitis model, isoniazid and rifampin treatment significantly reduced Mtb growth, and cotreatment with teriparatide did not change the anti-tuberculosis effect of isoniazid (INH) and rifampin (RFP). IP-10 and RANTES levels were significantly increased by Mtb infection, whereas teriparatide did not affect all chemokine levels as inflammatory markers. ALP and ARS staining indicated that teriparatide promoted osteoblastic function even with Mtb infection. Cotreatment with teriparatide and the anti-tuberculosis drugs activated bone formation (ALP-positive area increased by 705%, *P* = 0.0031). Teriparatide was effective against Mtb-infected MG63 cells without the anti-tuberculosis drugs (ARS-positive area increased by 326%, *P* = 0.0037). Teriparatide had no effect on the efficacy of anti-tuberculosis drugs and no adverse effect on the activity of Mtb infection in osteoblasts. Furthermore, regulation of representative osteoblastic inflammatory chemokines was not changed by teriparatide treatment. In the in vitro Mtb-infected MG-63 cell model of tuberculous spondylitis, cotreatment with the anti-tuberculosis drugs and teriparatide increased osteoblastic function.

## Introduction

Globally, an estimated 10 million people contracted tuberculosis in 2019^1^ and the proportion of extrapulmonary tuberculosis cases is increasing in developed countries^2^. Tuberculous spondylitis accounts for 1–5% of tuberculosis cases and represents ~ 50% of all bone and joint tuberculosis^3^. Compared to bacterial spondylitis, the diagnosis of tuberculous spondylitis is often delayed, and the worst complications, spinal deformities with bone destruction and neurological deficits, often follow^4^. Despite advances in diagnostic techniques, even in developed countries, neurological deficits are still present at the time of diagnosis in 45% of the cases^5^.

As anti-tuberculosis drugs are long-term drugs, external immobilization and bed rest are the conventional recommendations for patients with tuberculous spondylitis with pathologic fracture. However, treatment with only anti-tuberculosis medications for an extended period of up to 1 year is an inadequate strategy to address the catastrophic bone destruction. Furthermore, concurrent spinal surgery such as screw fixation and bone fusions still lead to poor surgical outcomes compared with that of general spinal surgery because of osteolysis and deterioration of bone formation due to inflammation^6^.

Therefore, traditional anti-tuberculosis agents may need to be combined with drugs that minimize the destruction of vertebrae and promote osseointegration to effectively treat tuberculosis infection. To fill this therapeutic gap, teriparatide, a recombinant human parathyroid hormone (PTH), could be considered as an optimal combination as an anabolic agent. However, no studies have been conducted on the safety and efficacy of teriparatide in patients who require anti-tuberculosis treatment.

Based on previous studies, teriparatide is recognized to be contraindicated in osteosarcoma or malignant melanoma because it could cause disease progression^7,8^, but to the best of our knowledge, its propensity to cause a similar problem in tuberculosis has not been determined. Minimal evidence for considering the clinical use of teriparatide for tuberculous spondylitis would come from the verification of two required characteristics. Teriparatide must not interfere with the action of anti-tuberculosis drugs and should not increase the rate of *Mycobacterium tuberculosis* (Mtb) infection; in the present study, we aimed to obtain experimental evidence to confirm that teriparatide meets the two requirements. Furthermore, we established an in vitro spondylitis model to evaluate whether the anabolic activity of teriparatide mediates its effects on Mtb-infected osteoblasts.

## Results

### Drug interaction: anti-tuberculosis drugs and teriparatide

**Figure 1:**
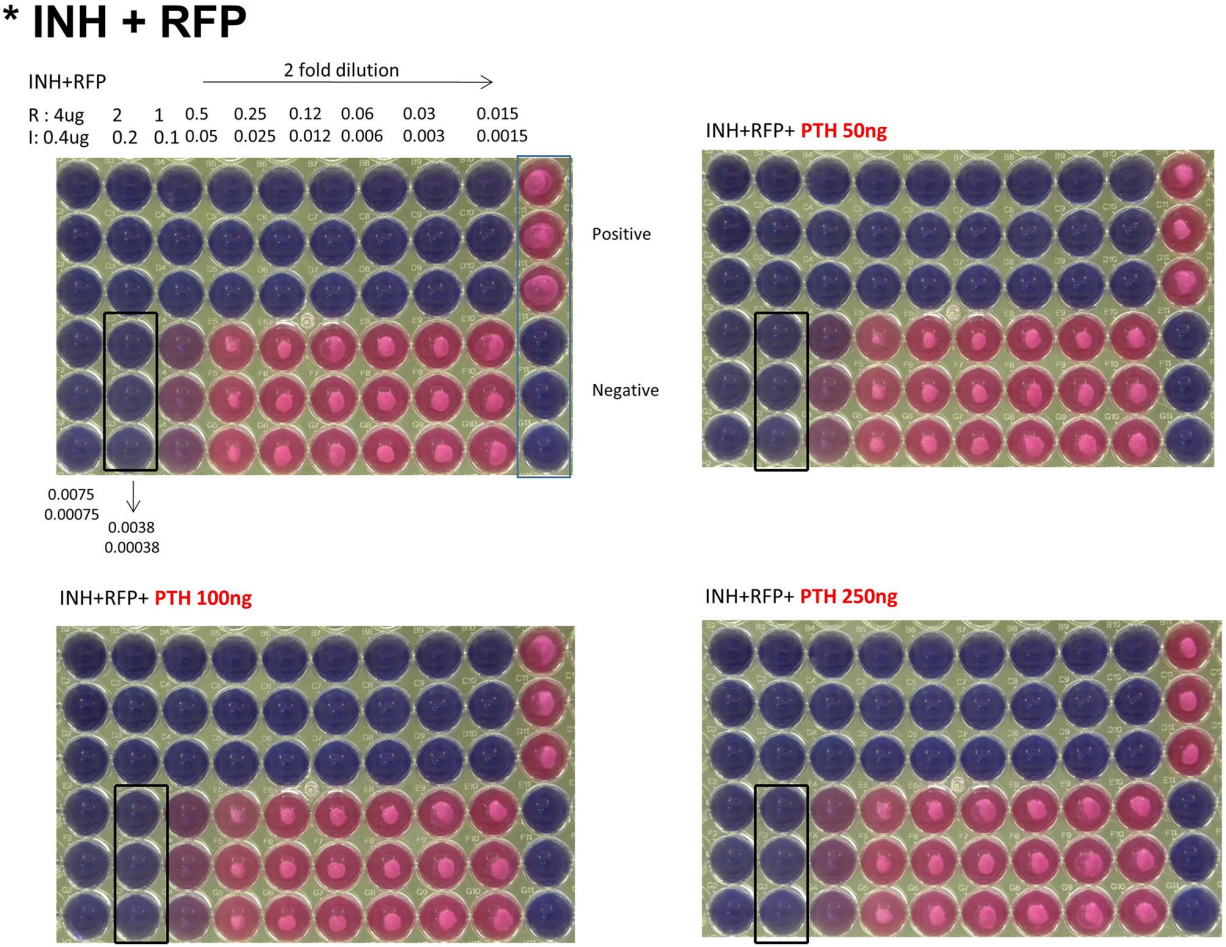
Minimum inhibitory concentration (MIC) assay of isoniazid plus rifampicin (INH + RFP) according to various teriparatide concentration (0, 50, 100, and 250 ng). Abbreviations: INH, isoniazid; PTH, teriparatide; RFP, rifampin.

The MICs of INH and RFP against the Mtb strain were confirmed to be 0.0038 and 0.00038 µg/mL, respectively, both alone and following cotreatment with teriparatide 50, 100, and 250 ng/mL (Fig. 1), indicating that teriparatide (0–250 ng/mL) showed no concentration-dependent effect.

### Effects of teriparatide on Mtb activity

**Figure 2:**
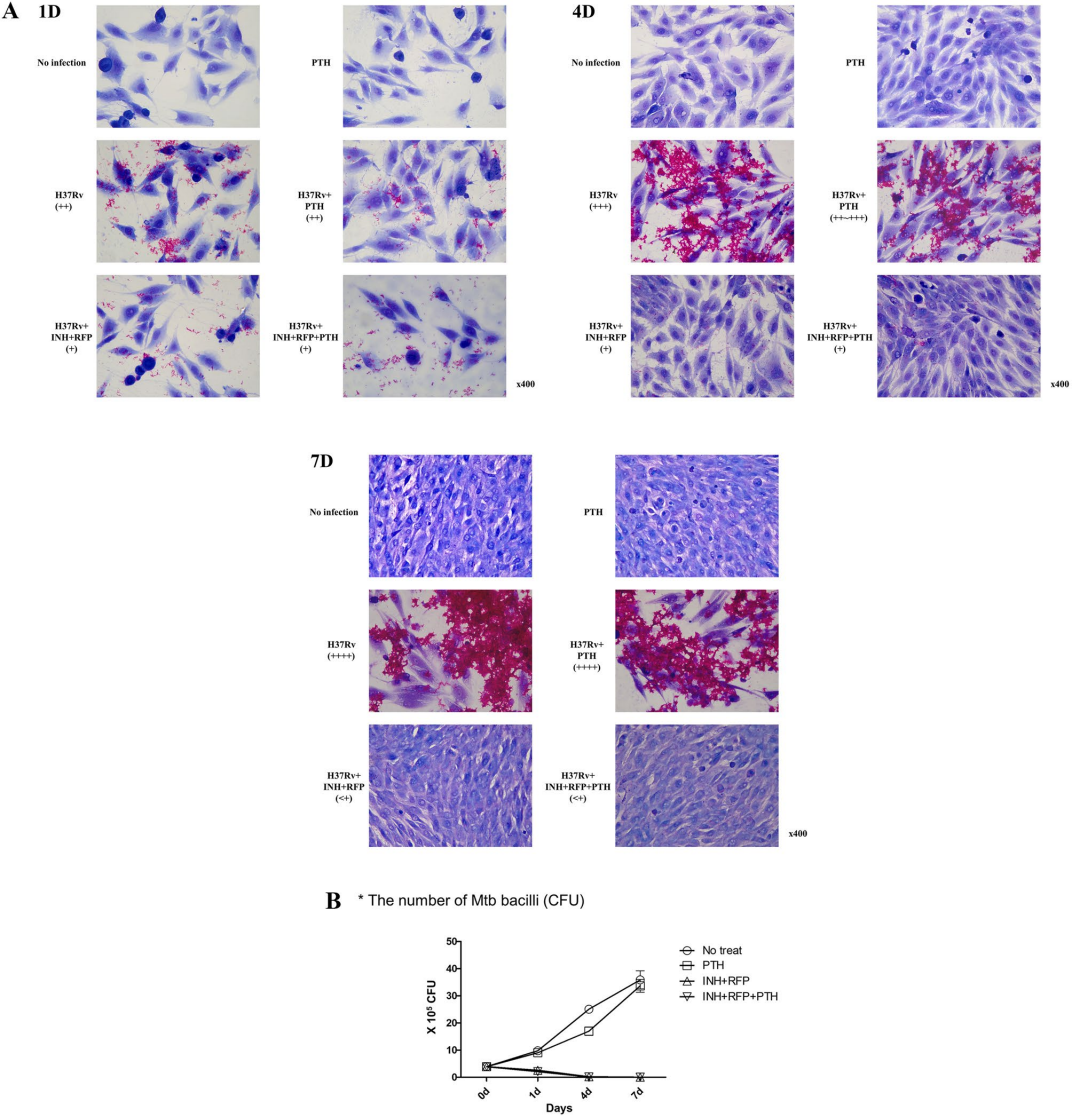
Microscopic images of acid fast bacillus (AFB) and Giemsa staining results. (**A**) + : < 500 bacili, +  + : 500–1000 bacilli, +  +  + : 1000–3000 bacilli, +  +  +  + : > 3000 bacilli). 400 × magnification. (**B**) CFU results. (**A**), 1 day after drug administration (1D). Anti-tuberculosis drugs showed slight inhibitory effect on Mtb growth, 4 days after drug administration (4D). Growth of Mtb increased, and anti-tuberculosis drugs significantly inhibited Mtb activity. No apparent difference was observed in osteoblast density according to concentration of teriparatide administered, 7 days after drug administration (7D). Significant proliferation of osteoblasts was evident. Mtb proliferation was significantly increased in the absence of anti-tuberculosis drugs. Anti-tuberculosis drugs completely inhibited bacterial growth. No apparent difference was observed in osteoblast density according to teriparatide administration; (**B**), CFU results by time point. Mtb activity of infected MG-63 increased four-fold for 7 days. Mtb was eradicated by anti-tuberculosis drug. Teriparatide did not affect Mtb activity. Abbreviations: AFB, acid-fast bacilli; CFU, colony-forming units; INH, isoniazid; PTH, teriparatide; RFP, rifampin; Mtb, *Mycobacterium tuberculosis*.

The results of the MFI and CFU evaluation of MG-63 cells infected with Mtb H37Rv and cultured for 7 days showed that the number of infecting Mtb bacilli significantly increased, from day 0 to day 7 (Table 1). Treatment with the anti-tuberculosis drugs eradicated the Mtb infection. Figure 2 shows the infection and growth of Mtb in MG63 cells during the in vitro tuberculous spondylitis model establishment. The results showed that cotreatment with teriparatide (50 ng/mL) did not adversely affect the anti-tuberculosis activity of INH or RFP, which showed no change in activity against Mtb (Table1, Fig. 2).

**Table 1:** Mean fluorescence intensity (MFI) and colony-forming units (CFU) results.

FACS (MFI)	H37Rv					H37Rv + PTH					H37Rv + INH + RFP					H37Rv + INH + RFP + PTH						
MFI	1	2	3	AVE	SEM	1	2	3	AVE	SEM	1	2	3	AVE	SEM	1	2	3	AVE	SEM		
Day 0	13.55	15.26	16	14.9	0.7	13.55	15.26	16	14.9	0.7	13.55	15.26	16	14.9	0.7	13.55	15.26	16	14.9	0.7		
Day 1	29.81	30.26	34.36	31.5	1.4	32.91	29.14	27.78	29.9	1.5	12.78	11.97	12.03	12.3	0.3	13.23	11.64	10.71	11.9	0.7		
Day 4	46.05	45.21	44.79	45.4	0.4	39.6	38.28	40.43	39.4	0.6	4.48	6.33	7.64	6.2	0.9	6.52	7.49	7.4	7.1	0.3		
Day 7	95.51	97.36	81.15	91.3	5.1	105.41	96.46	99	100.3	2.7	4.11	4.47	3.2	3.9	0.4	4.14	3.55	3.93	3.9	0.2		

CFU	H37Rv											H37Rv + PTH										
(× 10^6 cells)	1	2	3	4	5	6	7	8	9	AVE	SEM	1	2	3	4	5	6	7	8	9	AVE	SEM
Day 0	2.6	1.7	3.3	5.4	4.6	2.6	6	4.4	4.4	3.9	0.5	2.6	1.7	3.3	5.4	4.6	2.6	6	4.4	4.4	3.9	0.5
Day 1	10.2	9.2	10	8.8	9.2	10	6.4	15.2	8.8	9.8	0.8	7.2	6	9.2	9.2	7.2	11.2	10.4	10.4	10.4	9.0	0.6
Day 4	26.4	26.8	24.4	29.6	22.4	16.8	31.2	25.2	22.8	25.1	1.4	14.4	18.4	20	20.8	16	22.4	20.4	10.8	9.6	17.0	1.5
Day 7	52	51.2	35.2	34.8	37.2	31.2	27	31.5	22.5	35.8	3.3	20	42.4	30.4	37.2	43.2	33.6	30	30	36	33.6	2.4

CFU	H37Rv + INH + RFP											H37Rv + INH + RFP + PTH										
(× 10^6 cells)	1	2	3	4	5	6	7	8	9	AVE	SEM	1	2	3	4	5	6	7	8	9	AVE	SEM
Day 0	2.6	1.7	3.3	5.4	4.6	2.6	6	4.4	4.4	3.9	0.5	2.6	1.7	3.3	5.4	4.6	2.6	6	4.4	4.4	3.9	0.5
Day 1	1.95	1.85	1.95	4	4.1	3.7	1.8	1.4	2.4	2.6	0.4	1.6	1.85	1.9	2.3	1.9	3.4	1	2.4	2.4	2.1	0.2
Day 4	0.188	0.203	0.224							0.2	0.0	0.1	0.076	0.141							0.1	0.0
Day 7	0.0264	0.0238	0.024	0.0535	0.044	0.0485				0.0	0.0	0.0118	0.0114	0.014	0.0215	0.02	0.026				0.0	0.0

### Effects of teriparatide on Mtb-infected MG-63 Cells

After several preliminary experiments, we identified that the effective dosing protocol for determining the optimal activity of teriparatide against MG-63 cell proliferation was intermittent exposure to the drug. We changed the differentiation medium after treatment with 400 ng/mL teriparatide for only 4 h every 48 h. The result of the alkaline phosphatase (ALP) and alizarin red S (ARS) staining obtained following this protocol showed that teriparatide enhanced the osteoblastic function with or without eradicating Mtb.

**Figure 3:**
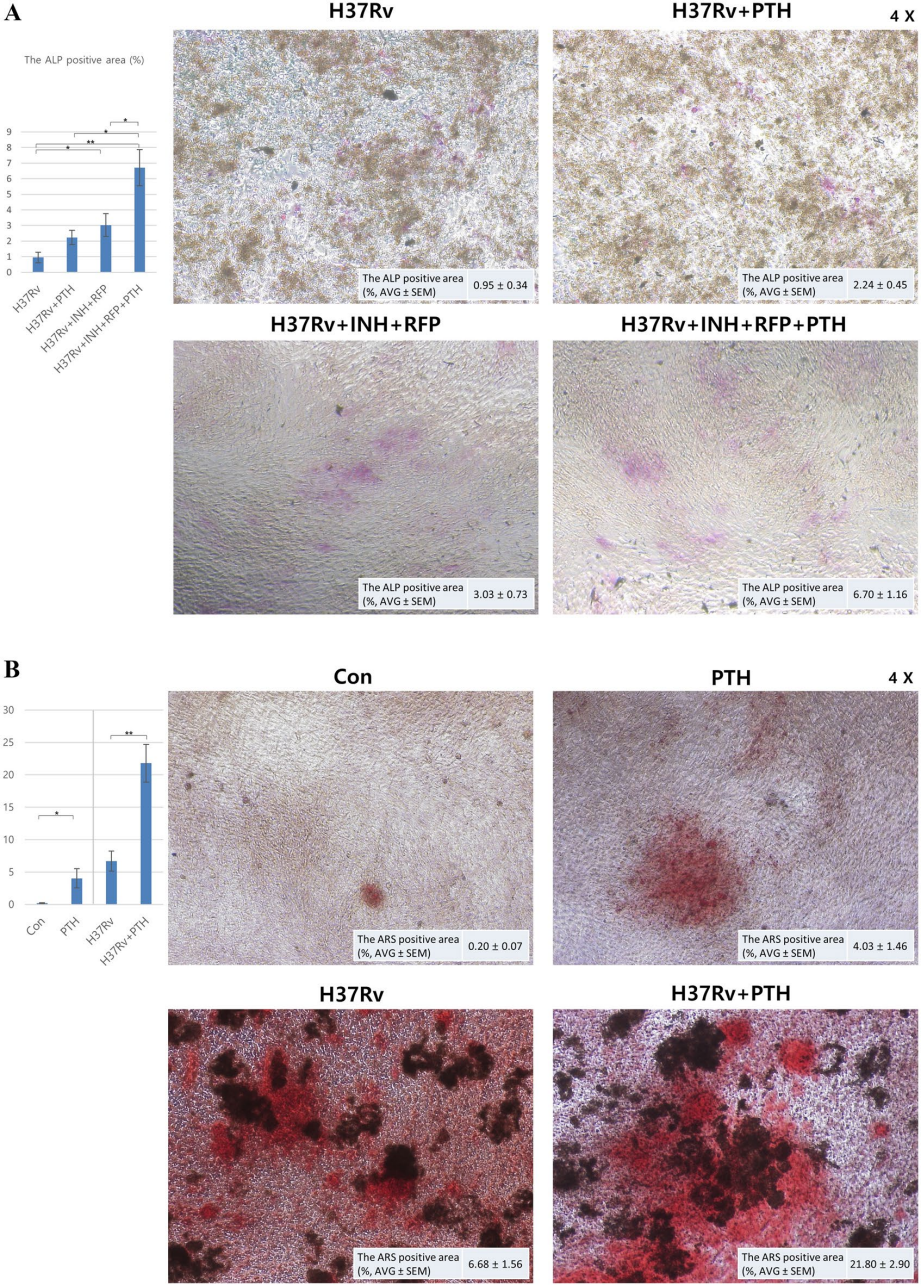
Alkaline phosphatase (ALP) and alizarin red S (ARS). (**A**) ALP- and (**B**) ARS-positive area was measured using bioquantification software (Bioquant Osteo II, R&M Biometrics, Nashville, TN, USA), and graphs show comparison of positive area for each group. Abbreviations: AVG, average; Con, control; INH, isoniazid; PTH, teriparatide; RFP, rifampin; SEM, standard error of mean; *P < 0.05; **, *P* < 0.01.

The ALP staining results on day 7 showed that eradication of Mtb with INH + RFP increased the ALP-positive area induced by teriparatide treatment by 705%, from 0.95 ± 0.34 to 6.70 ± 1.16 (*P* = 0.0031). The ALP positive area was higher following cotreatment with teriparatide and the anti-tuberculosis drugs (teriparatide with INH + RFP: 6.70 ± 1.16, *P* = 0.0116) than it was with teriparatide alone (2.24 ± 0.45). The ALP-positive area increased by 299%, from 2.24 to 6.70, by INH and RFP under the same teriparatide dosing condition (Table 2, Fig. 3A).

**Table 2:** Alkaline phosphatase (ALP) staining results.

ALP-positive area (%)	H37Rv	H37Rv + PTH	H37Rv + INH + RFP	H37Rv + INH + RFP + PTH
Test 1	2.12	1.84	4.10	6.04
Test 2	0.65	1.96	2.19	8.04
Test 3	0.60	1.38	1.09	3.27
Test 4	0.42	3.76	4.72	9.45
AVE	0.95	2.24	3.03	6.70
SEM	0.34	0.45	0.73	1.16
*P*-value (vs H37Rv)		0.0637	0.0415	0.0031
*P*-value (vs H37Rv + PTH)			0.3923	0.0116
*P*-value (vs H37Rv + INF + RFP)				0.0364

The ARS staining results on day 28 showed that teriparatide treatment increased the positively stained area of uninfected MG-63 cells (control) by 2015%, from 0.20 ± 0.07 to 4.03 ± 1.46 (*P* = 0.0398). The results of the control group in this study confirmed the validity of our ARS experimental protocol for evaluating the effect of teriparatide on MG63, and showed that it promoted osteogenesis.

The non-specific staining of Mtb and MG-63 cell necrosis indicated that the ARS-positive area was larger 28 days after Mtb infection progressed than it was in the absence of Mtb infection (Control: 0.20 ± 0.07, H37Rv: 6.68 ± 1.56, *P* = 0.006). Thus, the effect of teriparatide was investigated using the same protocol used for the control and the ARS staining was performed under Mtb infection conditions. Teriparatide treatment increased the ARS-positive area of Mtb-infected MG-63 cells by 326%, from 6.68 ± 1.56 to 21.80 ± 2.90 (*P* = 0.0037), and it effectively promoted osteogenesis in Mtb-infected MG-63 cells without Mtb eradication (Table 3, Fig. 3B).

**Table 3:** Alizarin red S (ARS) staining result.

ARS-positive area (%)	Control	PTH	H37Rv	H37Rv + PTH
Test 1	0.23	1.41	5.04	21.41
Test 2	0.06	0.81	5.75	15.91
Test 3	0.09	7.07	11.97	31.26
Test 4	0.40	6.83	3.97	18.61
AVE	0.20	4.03	6.68	21.80
SEM	0.07	1.46	1.56	2.90
*P* value (vs Con)		0.0398	0.006	0.0003
*P* value (vs PTH)			0.2612	0.0016
*P* value (vs H37Rv)				0.0037

### Regulation of chemokine secretion in Mtb-infected MG-63 Cells

**Figure 4:**
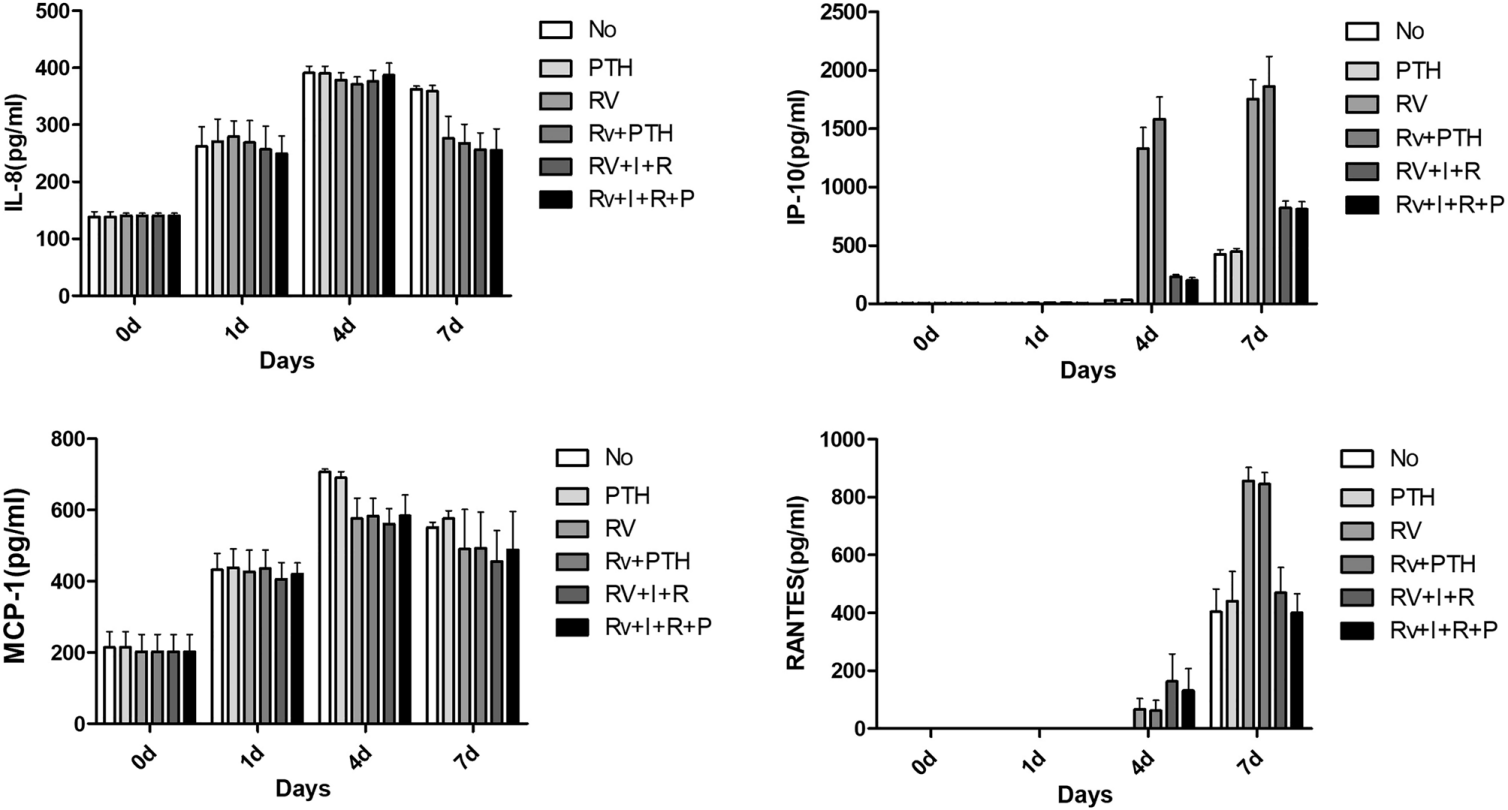
Enzyme-linked immunosorbent assay (ELISA) results of four representative chemokines related to pathologic inflammatory conditions in osteoblasts. Abbreviations: INH, isoniazid; IL-8, interleukin-8; IP-10, interferon γ-induced protein-10; MCP-1, monocyte chemoattractant protein-1; P, teriparatide; PTH, teriparatide; RIF, rifampin; RANTES, regulated upon activation normal, T cell expressed and presumably secreted; RV, *Mycobacterium tuberculosis* H37RV strain infection.

The levels of chemokines secreted following Mtb infection were measured and the results showed that teriparatide did not significantly affect secretion levels of the four chemokines investigated. These results suggest that teriparatide did not affect the inflammatory level of Mtb-infected osteoblasts. The levels of IL-8 and MCP-1 showed no difference among the experimental groups and were independent of the presence or absence of Mtb infection or administration of the anti-tuberculosis drugs and teriparatide. In contrast, Mtb infection induced significantly higher levels of IP-10 and RANTES than those of the uninfected group on days 4 and 7, whereas the anti-tuberculosis drugs significantly reduced these levels compared to those of the untreated group (Table 4, Fig. 4).

**Table 4:** Enzyme-linked immunosorbent assay (ELISA) of inflammatory chemokines.

	No (Control)						PTH						H37Rv					
	1	2	3	4	AVE	SEM	1	2	3	4	AVE	SEM	1	2	3	4	AVE	SEM
**IL-8(pg/ml)**																		
Day 0	151.1	144	120.6		138.57	9.21	151.1	144	120.6		138.57	9.21	142.3	147	132.7		140.67	4.21
Day 1	206.7	197.3	325.6	319.1	262.18	34.82	205.7	199.4	337.9	339.9	270.73	39.38	232.7	231.5	329.6	324.2	279.50	27.39
Day 4	364.6	377.1	402.5	419.2	390.85	12.31	386.7	360.2	419.7	394.9	390.38	12.26	362.4	350.4	405	395.6	378.35	13.05
Day 7	367.6	348.6	373.6	361.6	362.85	5.34	350.4	338.2	385.7	361.6	358.98	10.11	208.8	210	337.5	349.5	276.45	38.79
**MCP-1(pg/ml)**																		
Day 0	163.07	178.25	302.2		214.51	44.07	163.07	178.25	302.2		214.51	44.07	150.57	159.05	297.6		202.41	47.66
Day 1	503.34	520.36	345.3	359.1	432.03	46.30	551.13	501.88	351.7	349.4	438.53	51.78	530.75	531.68	328.6	316	426.76	60.36
Day 4	729.34	694.11	698.7	706.3	707.11	7.82	730.95	707.52	655.1	667.8	690.34	17.55	477.29	475.77	669.2	682.5	576.19	57.60
Day 7	550.77	515.54	587.7	549.5	550.88	14.74	552.38	528.95	614.7	609.6	576.41	21.21	298.71	297.2	688.9	676.6	490.35	111.11
**IP-10(pg/ml)**																		
Day 0	3	2.4	4.5		3.30	0.62	3	2.4	4.5		3.30	0.62	2.5	2.9	5		3.47	0.78
Day 1	2.6	4.9	5	6.4	4.73	0.79	1.2	2	7.5	7.3	4.50	1.68	10.4	6.2	5.2	3.9	6.43	1.41
Day 4	24.7	24.6	33.5	40.1	30.73	3.76	25.3	28.3	40.3	38.9	33.20	3.76	1021.4	1025.8	1683.9	1593	1331.03	178.46
Day 7	353.4	359.3	488	494.6	423.83	39.00	401.6	394.1	494.8	493.5	446.00	27.84	1460.9	1467.8	2087.5	1995.3	1752.88	167.65
**RANTES(pg/ml)**																		
Day 0	0	0	0	0	0.00	0.00	0	0	0	0	0.00	0.00	0	0	0	0	0.00	0.00
Day 1	0	0	0	0	0.00	0.00	0	0	0	0	0.00	0.00	0	0	0	0	0.00	0.00
Day 4	0	0	0	0	0.00	0.00	0	0	0	0	0.00	0.00	139.6	123.9	0	0	65.88	38.17
Day 7	555.2	526.3	277.14	255.12	403.44	79.62	611.7	622.8	243.36	282.86	440.18	102.57	952.3	920.4	764.5	786.77	855.99	47.07

	H37Rv + PTH						H37Rv + INH + RFP						H37Rv + INH + RFP + PTH					
	1	2	3	4	AVE	SEM	1	2	3	4	AVE	SEM	1	2	3	4	AVE	SEM
**IL-8(pg/ml)**																		
Day 0	142.3	147	132.7		140.67	4.21	142.3	147	132.7		140.67	4.21	142.3	147	132.7		140.67	4.21
Day 1	200.2	205.2	320.9	349.1	268.85	38.64	188.8	185	324.3	330	257.03	40.51	198.6	193.3	302.3	303.6	249.45	30.91
Day 4	338.3	362.4	387.4	395.3	370.85	12.92	339.9	346	408.8	411.8	376.63	19.49	342.2	359.9	424	423	387.28	21.23
Day 7	211.9	208.8	325.4	325.4	267.88	33.22	205.6	205.7	313.4	301.3	256.50	29.46	186.3	197.5	301.3	337.5	255.65	37.61
**MCP-1(pg/ml)**																		
Day 0	150.57	159.05	297.6		202.41	47.66	150.57	159.05	297.6		202.41	47.66	150.57	159.05	297.6		202.41	47.66
Day 1	500.34	547.88	347.4	347.2	435.71	51.95	472.71	499.11	317	333.2	405.51	46.85	482.21	466.48	371.4	360.5	420.15	31.53
Day 4	501.57	494.55	662.4	674.3	583.21	49.24	489.07	481.98	644	628.2	560.81	43.61	469.09	495.73	685	685	583.71	58.73
Day 7	323	315.98	695.9	637.5	493.10	100.95	310.5	303.41	623.2	586	455.78	86.27	290.52	317.16	679.3	667.4	488.60	106.83
**IP-10(pg/ml)**																		
Day 0	2.5	2.9	5		3.47	0.78	2.5	2.9	5		3.47	0.78	2.5	2.9	5		3.47	0.78
Day 1	17.1	10.2	4.3	4.4	9.00	3.03	9	11.4	5.3	7.5	8.30	1.28	4.8	5.6	3	6.2	4.90	0.70
Day 4	1252.7	1249.1	1865.7	1956.6	1581.03	191.50	196.3	195.7	264.4	261.1	229.38	19.28	155	155.9	247.4	237.6	198.98	25.21
Day 7	1412.8	1410.1	2257.6	2353.4	1858.48	258.83	937.9	909.1	718.9	715.6	820.38	59.83	908.4	931.8	701.9	692.2	808.58	64.60
**RANTES(pg/ml)**																		
Day 0	0	0	0	0	0.00	0.00	0	0	0	0	0.00	0.00	0	0	0	0	0.00	0.00
Day 1	0	0	0	0	0.00	0.00	0	0	0	0	0.00	0.00	0	0	0	0	0.00	0.00
Day 4	127.6	120.9	0	0	62.13	35.89	332	321.3	0	0	163.33	94.32	262.2	263	0	0	131.30	75.81
Day 7	927.1	872.2	845.5	737.86	845.67	39.75	635.4	605.1	321	317.32	469.71	87.14	532.4	493.6	310.5	264.47	400.24	66.25

IL-8, interleukin-8; IP-10, interferon γ-induced protein 10 kDa; MCP-1, monocyte chemoattractant protein-1;RANTES, regulated upon activation, normal T cell expressed and presumably secreted.

## Discussion

To the best of our knowledge, the effectiveness of cotreatment with anti-tuberculosis agents and teriparatide has not been investigated, and therefore, we aimed to examine this phenomenon in this study. First, our in vitro study revealed that teriparatide did not affect the efficacy of anti-tuberculosis drugs. Second, we established an in vitro model of Mtb infected osteoblast using H37rv with MG-63, and demonstrated that teriparatide did not increase the rate of Mtb infection. In addition, we verified that teriparatide treatment increased the anabolic bone effect in infected and uninfected (control) osteoblasts.

Clinicians have implemented anti-tuberculosis drugs as the first-line therapy for spondylitis treatment. Furthermore, eradication of Mtb using anti-tuberculosis agents is the most important factor to restore the osteogenic ability of infected osteoblasts. The first requirement for teriparatide to ensure its suitability for use in combination therapy is that it should not reduce the effectiveness of the anti-tuberculosis drugs. The in vitro resazurin MIC method showed that teriparatide did not adversely affect the anti-tuberculous effect of INF and RFP, which eliminated concerns about drug–drug interactions.

The data on optimal drug regimens and duration for tuberculous spondylitis treatment are limited because of drug-resistant Mtb. However, an observational study showed that spinal lesions contain fewer bacilli than do pulmonary lesions and are less likely to have drug-resistant mutants^9^. The two-drug regimen of only INH and RFP in combination was used in this study, based on the design of two previously reported randomized controlled trials for tuberculous spondylitis treatment^10,11^. Clinical trials of the short chemotherapy course for tuberculous spondylitis in Hong Kong, India, and South Korea reported that regimens using INH and RFP produced comparable results with those that used INH with either ethambutol or para-aminosalicylic acid^11^.

The recombinant human PTH analog teriparatide was approved in 2002 by the US Food and Drug Administration (FDA) for the treatment of osteoporosis at high risk for fracture and increased bone mass in postmenopausal women^12^. This drug has been demonstrated to promote bone healing and prevent fragility fractures in both rats and humans^13,14^. In addition to its numerous clinical advantages, teriparatide has some less well‑known contraindications, such as a history of radiation therapy^15^, the presence of primary malignant and metastatic bone tumors^7,16^, and Paget's disease^17^. As tuberculous spondylitis might also be a contraindicated comorbidity, targeted experiments to determine whether teriparatide enhances infection and inflammation induced by Mtb infection is expedient. In the present study, we showed that teriparatide did not increase the activity of Mtb based on the CFU analysis of the infected MG-63 cell line. Furthermore, the ELISA results revealed that teriparatide lacked regulatory effects on the selected chemokines known to be related to changes in bone metabolism due to osteomyelitis or bone tumor. This observation indicated that teriparatide was not associated with changes in inflammation of the Mtb-infected osteoblasts.

In vivo production of chemokines during human Mtb infection is well documented^18,19^. However, studies of osteoblasts in osteomyelitis are relatively few. We evaluated some chemokines with the most established role in physiological and pathological bone remodeling^20^. For inflammatory osteomyelitis, stimulation with tumor necrosis factor (TNF)-α and IL-1β has shown that osteoblasts secrete IL-8^21^, MCP-1^22,23^, and IP-10^24^. Our results showed that IL-8 and MCP-1 were increased under all conditions regardless of Mtb infection and their levels peaked on day 4 after infection, followed by a decrease on day 7. Mtb infection did not contribute to increasing levels of the two inflammatory chemokines, IL-8 and MCP-1. A role for IL-8 in bone metastatic disease was demonstrated in studies with breast cancer cells^25,26^. Osteoblasts and osteoclasts were shown to express IL-8 following stimulation with inflammatory mediators^27^. IL-8 stimulates bone resorption and could act as an essential regulatory signal for pathological bone remodeling. Moreover, the pro-inflammatory chemokine MCP-1, which plays a crucial role in bone remodeling^28^, was shown to be involved in several pathological conditions. For instance, MCP-1 contributed to bone metastasis development in cancers such as multiple myeloma^29^, prostate cancer^30^, oral squamous cell carcinoma^31^, and breast cancer^32^. MCP-1 increases tumor growth and bone metastasis by recruiting macrophages and osteoclasts to the tumor site^33^. Moreover, inflammatory mediators or bacteria were found to induce the expression of MCP-1 by osteoblasts in vivo^34,35^, which contributed to inflammatory bone loss. In our experiment, IL-8 and MCP-1 did not increase specifically with Mtb infection; therefore, a difference in chemokine expression likely exists between the diseases mentioned above and tuberculous spondylitis.

In contrast, the secretion of IP-10 and RANTES, which were specifically increased by Mtb infection, significantly decreased after anti-tuberculous drug treatment. Numerous studies attempted to characterize the expression of chemokines in response to Mtb in vitro and in vivo, in human and murine systems. However, synthesizing an understanding of cell infiltration and granuloma formation using results from these descriptive studies has proved difficult^36^. Human macrophages produced RANTES in response to virulent stains of M. tuberculosis^37^. Studies performed using bronchoalveolar lavage fluid indicate elevated levels of RANTES and IP-10 in tuberculosis patients compared to uninfected controls^38–40^. Expression of IP-10 in response to M. tuberculosis infection in mice has been reported^41,42^.

Previous studies suggest that RANTES is involved in the pathological progression of rheumatoid arthritis, osteoarthritis, osteomyelitis, and posttraumatic responses^43,44^. RANTES, along with other β-chemokines, has been shown to induce osteoclast chemotaxis^45^. IP-10 also promotes osteoclastic differentiation and osteolysis^46^, and impacts pathological bone remodeling in osteoporosis^47^, bone metastasis^48^, and rheumatoid arthritis^49^. The results of this study showed that the pathological microenvironment of tuberculous spondylitis could include RANTES and IP-10. Furthermore, IP-10 and RANTES are secreted later than MCP-1 and may be associated with delayed recruitment and activation of T lymphocytes during inflammation^43^. T lymphocyte recruitment is essential for granuloma development, and the production of IP-10 may be necessary for mediating the establishment of the type 1 cytokine profile observed in tuberculosis^18^.

Teriparatide has been widely demonstrated to reduce the risk of osteoporotic vertebral compression fractures. The primary motivation for this study was also the possibility of lowering the fracture risk of infectious spondylitis. Vertebral bodies of infectious spondylitis patients exhibit osteolytic change. A Japanese orthopedic surgeon, Shinohara, reported that vertebral body erosion was rapidly restored in pyogenic spondylitis by teriparatide therapy^50^. However, this was an off-label use of this agent without any evidence to support its usefulness in spondylitis.

Debilitating spontaneous destruction of bone and consequent severe spinal deformity are typical clinical courses of tuberculous spondylitis. In a clinical trial, teriparatide showed an effect on fracture healing^51^. Moreover, animal studies on fracture models have shown that the addition of teriparatide promotes fracture healing^52,53^. Compared to previously reported pathologic conditions such as severe osteoporosis, trauma, or pyogenic spondylitis, Mtb infection is a much more unfavorable microenvironment for bone healing. Surgical treatment is inevitable in cases where fracture healing fails and spinal deformity with neurologic deterioration eventually occurs. However, the success rate of complete bone fusion during anti-tuberculosis treatment could be unsatisfactory^6,10^. Teriparatide has the potential to improve the osteointegration outcome of spondylitis surgeries. Although in non-infectious conditions, multiple studies had indicated that daily administration of teriparatide might promote osseous union in patients with osteoporosis undergoing posterior fusion with or without interbody support^54,55^.

In addition, Kuroshima et al. reported positive effects of teriparatide in preventing osteonecrosis in rats administered bisphosphonates and steroids^56^. Moreover, osteoblasts were considerably increased and osteoclasts and necrotic bone were decreased by teriparatide treatment after bisphosphonates and steroid use^56^. Teriparatide had a positive impact on the resolution of the inflammatory response of bone healing, but the mechanism of action of teriparatide is unknown^57^.

The prevailing view for many years has been that osteoclasts do not express PTH receptors and that PTH’s effects on osteoclasts are mediated indirectly via osteoblasts^58^. However, teriparatide may have a direct effect on osteoclasts which reduces the bone resorption rate^58,59^. In addition, teriparatide reduce inflammatory cytokines, so it is likely to inhibit osteoclast-mediated bone resorption. In addition, teriparatide had an inhibitory effect on several types of acute inflammation, reported previously^60^. Dohke et al. demonstrated that teriparatide rapidly and significantly inhibited the expression of inflammatory cytokines, such as IL-1β, IL-6, and TNF-α in an OVX mouse model^61^. Inhibition of these inflammatory cytokines by teriparatide may have the effect of reducing the osteoclast differentiation. According to these studies, we estimate that the total amount of bone formation will be positive, even including bone resorption by osteoclasts.

Since osteoclast activity is increased in Mtb-infected Ob, the inhibition effect of inflammatory cytokines by teriparatide may have an adjuvant action to alleviate bone destruction in tuberculous spondylitis. Moreover, Mtb powder lysed by ultrasound significantly enhanced osteoclast formation, bone absorption, and facilitate secretion of TNF-α and IFN-γ. Furthermore, it significantly increased mRNA expression of receptor activator of nuclear factor κ-B ligand (RANKL) in osteoblast and simultaneously decreased osteoprotegerin (OPG) expression, thus decreasing OPG/RNAKL ratio^62^. OPG is an antagonist to osteoclast differentiation and inhibits the RANKL/RANK signaling pathway by binding to RANKL^63^.

To prove that teriparatide has an anabolic effect on bone under Mtb-infected osteoblasts, we used ALP and ARS staining methods. ALP staining was performed on the 7th day and ARS staining was used to detect the mineralization nodules on the 28th day after differentiation^64^. Using INH-RFP and teriparatide combination treatment, 705% (*P* = 0.0031) of osteogenesis promotion was achieved compared to the no-treatment group in the ALP staining. In the ARS staining, 326% (*P* = 0.0037) of osteogenesis promotion was achieved through teriparatide administration. Based on the present study, we expect teriparatide to improve the clinical outcome by reducing the risk of vertebral fractures with Mtb infection and increasing the success rate of both conservative and surgical treatment.

This study has several limitations that are worth mentioning. First, in the MIC test, we arbitrarily determined the dose of teriparatide without any experimental or clinical evidence. The experimental concentrations used should be extrapolated based on the dose to be used in humans. Second, although we showed that teriparatide improved osteoblastic function in an in vitro tuberculous spondylitis model, only bioquantification software was used in the evaluation without statistical analysis. The software used might have been insufficient for the high level of evidence of osteogenic activation of infected osteoblasts. Third, osteoblast culture under various environments would be required for more meaningful conclusions. Future studies should investigate the microscopic changes in osteoblasts under an infectious environment using a lower Mtb MOI than that used in this study or only the exudate secreted by Mtb should be treated. Culturing infected osteoblasts directly from a patient with tuberculous spondylitis should also be considered. Fourth, although osteoclasts have an important role in tuberculous spondylitis, we did not investigate osteoclasts in this study, mainly because they lack receptors for teriparatide. Consequently, our experiments in this study were performed only with osteoblasts. Further studies would be necessary to analyze the osteoblast-osteoclast coupling chemokines, RANKL, osteoprotegerin, and granulocyte colony-stimulating factor (G-CSF). Co-culture of both osteoblasts and osteoclasts would also be helpful in providing additional information. Fifth, drugs other than teriparatide, which have bone formation effects, were not evaluated in the present study. In contrast to teriparatide, denosumab is an anti-resorptive agent that reduces the development and activity of osteoclasts by inhibiting the RANKL signaling pathway. Moreover, romosozumab is a bone-forming agent that inhibits sclerostin to promote bone formation and suppress bone resorption through a so-called “dual-effect”^65,66^. These molecular-targeted drugs are prominent in the field of osteoporosis treatment^66^. It is necessary to compare the effectiveness of each drug for tuberculous spondylitis in future study.

In conclusion, the target of tuberculous spondylitis treatment is elimination of the source of infection by administering anti-tuberculosis drugs. Moreover, adding a bone anabolic agent to alleviate bone destruction should also be considered. Thus, we sought to establish an experimental rationale for cotreatment with anti-tuberculosis drugs and teriparatide. We found that teriparatide did not affect the efficacy of anti-tuberculosis drugs and had no adverse effect on the progression of Mtb infection in osteoblasts. Furthermore, it did not change the expression of osteoblastic inflammatory chemokines. Finally, we showed that the intrinsic osteogenesis-promoting activity of teriparatide was maintained even in Mtb-infected osteoblasts and these results might serve as additional compelling evidence to support the potential usefulness of cotreatment with anti-tuberculous drugs and teriparatide in tuberculous spondylitis.

## Methods

### Experimental design

**Figure 5:**
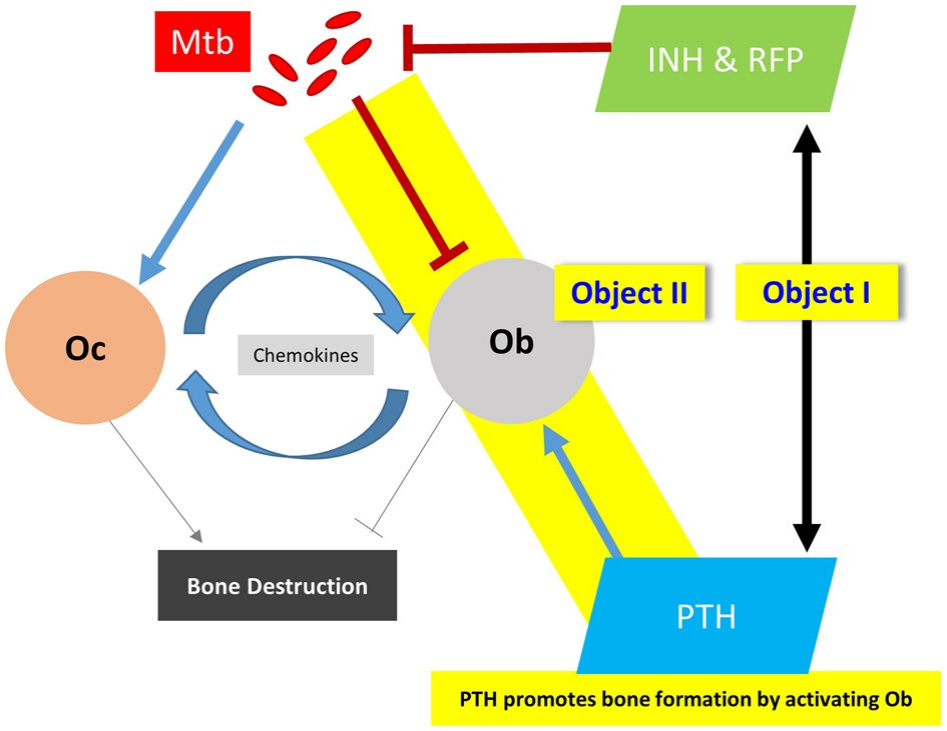
Schematic diagram of experimental design. Study objective was to address two questions to provide essential evidence to support the usefulness of teriparatide in patients with tuberculous spondylitis in clinical practice. Abbreviations: INH, isoniazid; Mtb, *Mycobacterium tuberculosis*; Ob, Osteoblast; Oc, Osteoclast; PTH, teriparatide; RFP, rifampin.

This study had two primary objectives (Fig. 5). First, we investigated whether teriparatide reduces the efficacy of anti-tuberculosis drugs. To this end, we used the virulent Mtb H37Rv strain (American Type Culture Collection [ATCC] 27,294; ATCC, Manassas, VA, USA) to evaluate the minimum inhibitory concentration (MIC) of the two most widely prescribed standard anti-tuberculosis agents, isoniazid (INH) and rifampin (RFP). This assay was conducted using the resazurin MIC method.

Second, an in vitro model of tuberculous spondylitis was established by infecting the human osteoblastic MG-63 cell line (KCLB no. 21427, Korean Cell Line Bank, Seoul, South Korea) with the Mtb H37Rv strain. This model was subsequently used to examine the effect of teriparatide on the infectious activity of Mtb. Levels of several chemokines were also measured using the enzyme-linked immunosorbent assay (ELISA). Finally, we evaluated the osteogenesis-promoting activity of teriparatide on Mtb-infected osteoblasts using alkaline phosphatase (ALP) and alizarin red S (ARS) staining.

### Resazurin MIC method

For INH and RFP (catalog nos. I3377 and R3501, respectively; Sigma-Aldrich, St. Louis, MO, USA), the drug powder samples were dissolved in distilled water (DW) and methanol, respectively, to a concentration of 0.5 mg/mL and stored at −20 ℃. Teriparatide (Eli Lilly and Company, Indianapolis, IN, USA) was dissolved in DW to a 5 g$\upmu$/mL solution and stored at −20 °C. Resazurin sodium salt (catalog no. R7017; Sigma-Aldrich, St. Louis, MO, USA) was dissolved in DW to a concentration of 0.025% immediately before the experiment. The Mtb H37Rv strain was cultured for 2 weeks in Middlebrook 7H9 broth containing 10% oleic albumin dextrose catalase (OADC), 0.5% glycerol, and 0.05% Tween 80 (Sigma-Aldrich, St. Louis, MO, USA).

**Figure 6:**
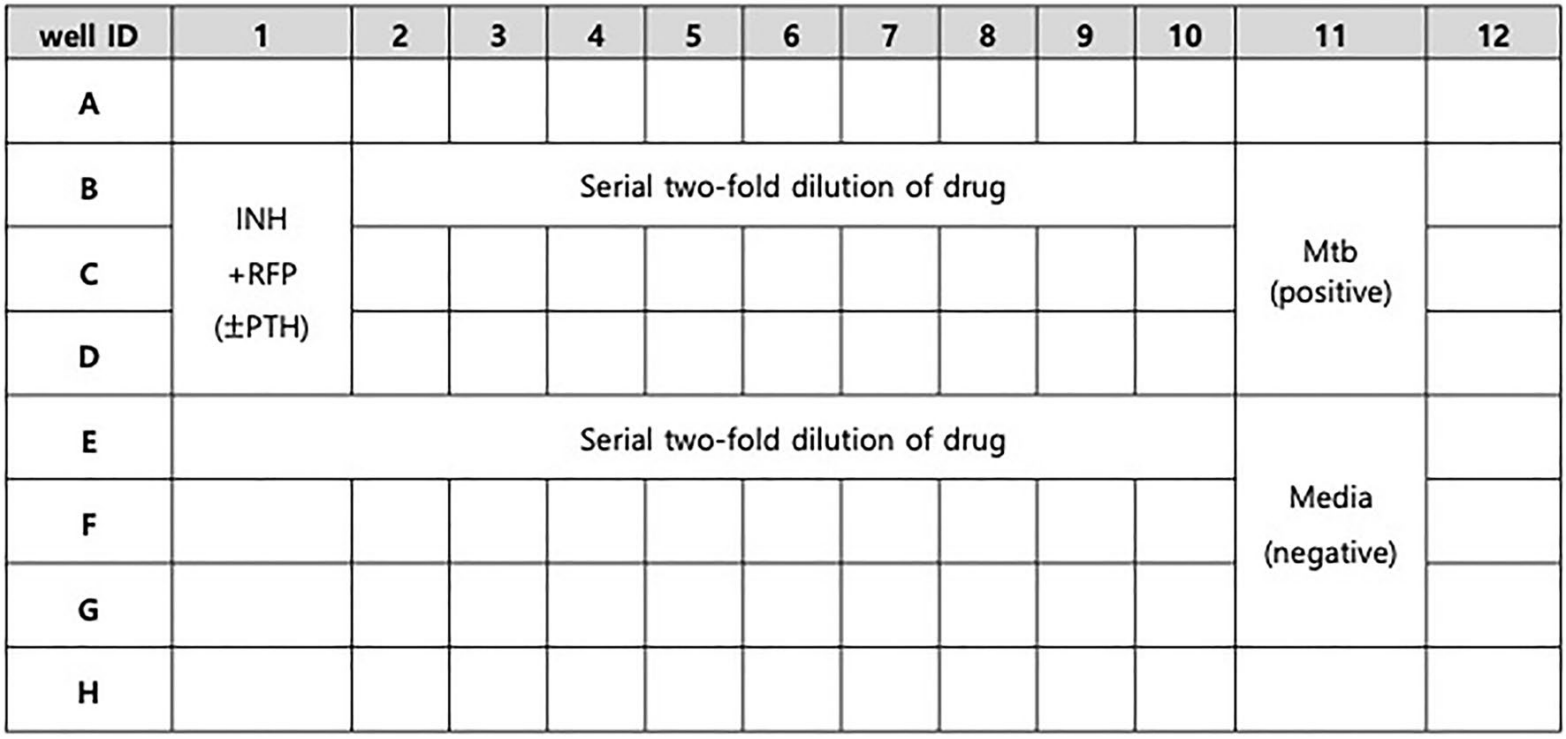
Image of 96-well plate (Corning ® 96 Well TC-Treated Microplates, Sigma-Aldrich, St. Louis, MO, USA) for resazurin minimum inhibitory concentration (MIC) assay method. Abbreviations: INH, isoniazid; Mtb, *Mycobacterium tuberculosis*; PTH, teriparatide; RFP, rifampin.

The test was performed as follows: (1) Culture medium (100 µL) was placed in a 96-well plate (Corning ® 96 Well TC-Treated microplates; Sigma-Aldrich, St. Louis, MO, USA). (2) Then,  µL each of 32 µg/mL RFP and 3.2 µg/mL INH solutions were added to the top well to obtain final concentrations of 4 µg/mL RFP and 0.4 µg/mL INH, followed by a twofold serial dilution. (3) Then, 100 µL bacterial solution was diluted to an optical density (OD) 0.01/mL. The Mtb bacilli were incubated with the anti-tuberculosis drugs for 10 days to establish the MIC in this experiment. (4) Predesignated wells were treated with teriparatide at concentrations of 50, 100, and 250 ng/mL. (5) The plates were wrapped in foil and incubated for 1 week in 5% CO_2_ incubator at 37 ℃. (6) After treatment with 20 µL of a 0.025% resazurin solution, the plates were incubated for 2 more days. (7) Finally, color development was examined and confirmed in the plates to determine the growth status of the cells: pink and blue, growth or no growth of Mtb, respectively (Fig. 6).

### MG-63 osteoblast culture

The human osteoblastic MG-63 cell line was obtained from the Korean Cell Line Bank (KCLB no. 21427) and maintained in Dulbecco’s modified Eagle’s medium ([DMEM]/high glucose; HyClone, Logan, UT, USA) supplemented with 10% fetal bovine serum (FBS; HyClone, Morningside, QLD, Australia) and 1% penicillin and streptomycin (P/S; Gibco, Invitrogen Corporation, Carlsbad, CA, USA) in a humidified 5% CO_2_ atmosphere at 37 °C. The MG-63 cells were trypsinized with 0.25% trypsin (HyClone, Logan, UT, USA), seeded, finally cultured for 48 h at an appropriate density, and were used when they achieved 90% confluence.

### Mtb H37Rv strain culture

The Mtb H37Rv strain was maintained in Middlebrook 7H9 medium with Tween 80, albumin, and glycerol for 14 days at 37 °C in an atmosphere of 5% CO_2_.

### Mtb-infected osteoblast: an in vitro model

**Figure 7:**
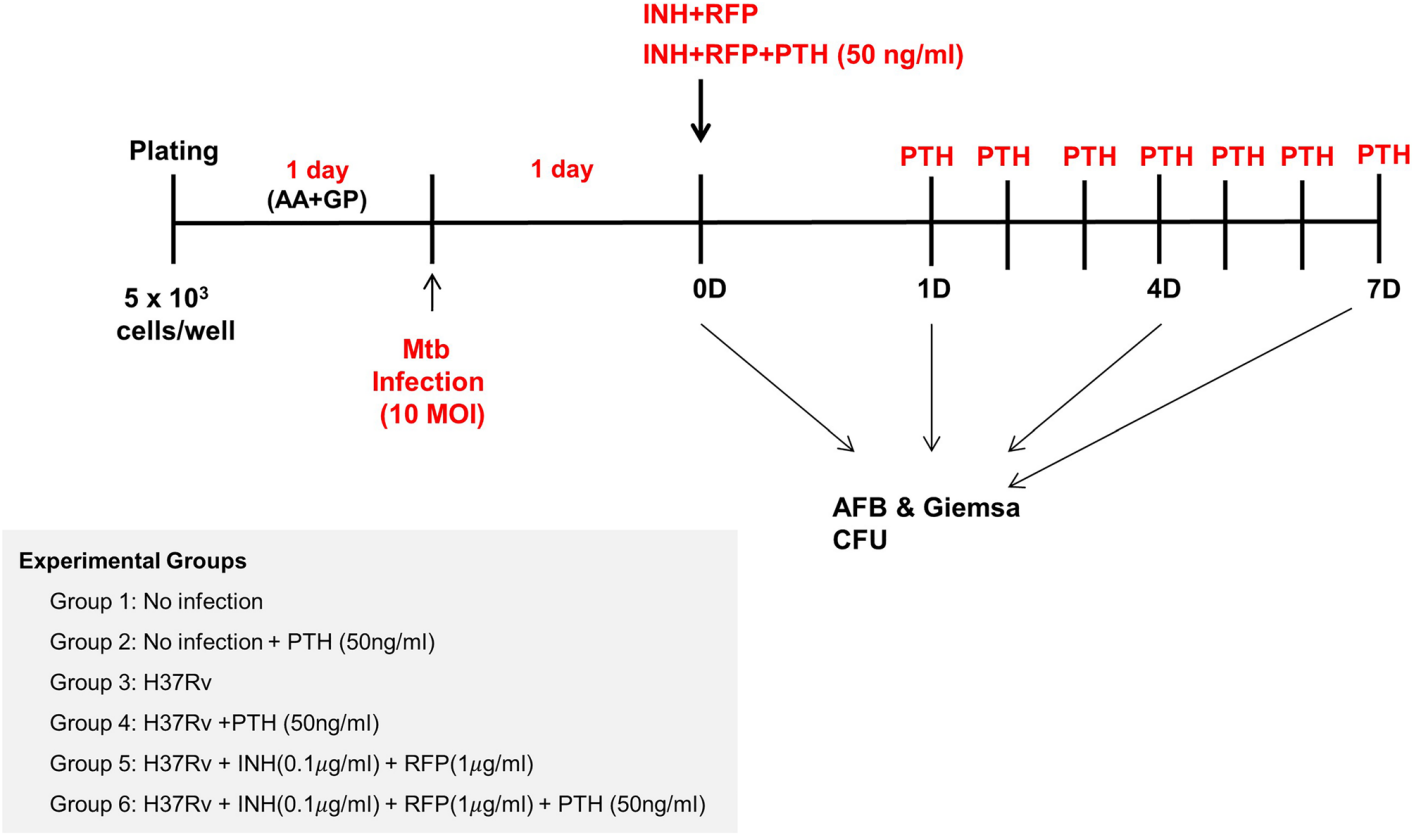
Experimental protocol for infecting MG-63 osteoblasts with *Mycobacterium tuberculosis* (Mtb) and cotreatment of cells with anti-tuberculosis drugs and teriparatide. Abbreviations: AA, ascorbic acid; AFB, acid-fast bacilli; CFU, colony-forming units; FACS, fluorescence-activated cell sorting; GP, $\upbeta$-glycerophosphate; INH, isoniazid; PTH, teriparatide; RFP, rifampin.

MG-63 cells were plated at a density of 5 × 10^3^ cells/well on medium mixed with DMEM, 10% FBS, ascorbic acid (50 µg/mL), and $\upbeta$-glycerophosphate (10 mM) and incubated for 24 h. The cells were transfected with the Mtb H37Rv strain at a rate of 10 multiplicity of infection (MOI), incubated overnight, and then each group was treated with the anti-tuberculosis drugs (INH and RFP) and teriparatide (50 ng/mL). On day 1, 4, and 7 post treatment, acid-fast bacilli (AFB) and Giemsa staining were performed. The established in vitro Mtb-infected MG-63 cell model was evaluated using colony-forming units (CFU) assessment (Fig. 7).

### Osteogenic activity of Mtb-infected osteoblasts

**Figure 8:**
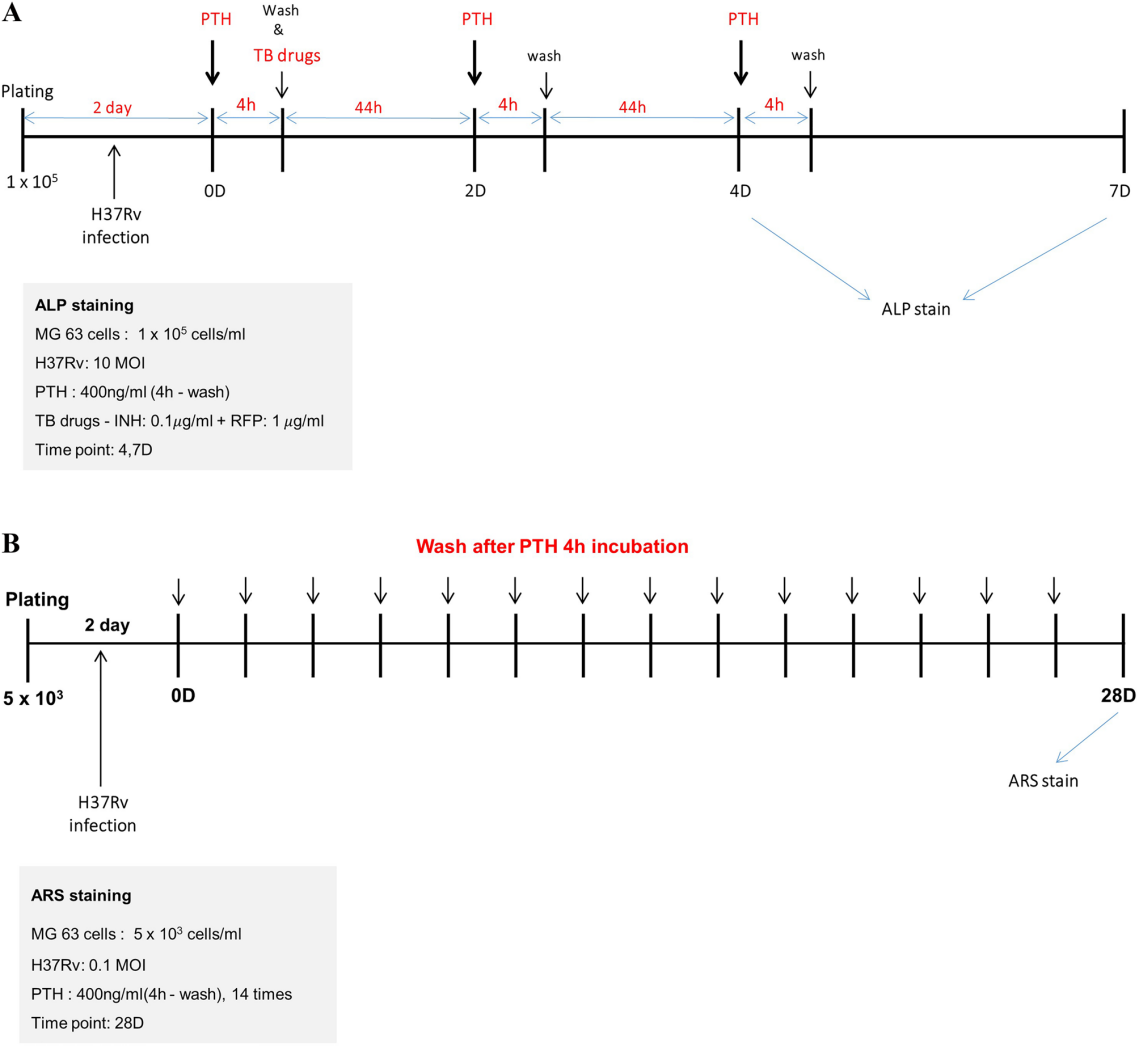
Experimental protocol for measuring osteoblastic activity of *Mycobacterium tuberculosis* (Mtb)-infected MG-63 cells. (**A**) ALP and (**B**) ARS staining. Abbreviations: ALP, alkaline phosphatase; ARS, Alizarin red S; INH, isoniazid; MOI, multiplicity of infection; PTH, teriparatide; RFP, rifampin; TB, anti-tuberculosis.

The preliminary experiment identified the teriparatide treatment protocol that induced the highest increase in osteoblastic activity of the MG-63 cells, which is shown in Fig. 8A. The cells were treated with teriparatide 400 ng/mL every 48 h, incubated for 4 h, washed, and then the culture medium was replaced. For ALP staining, MG-63 cells were plated at a density of 1 × 10^5^ cells/well with 10 MOI Mtb on medium and the Stem TAG™ ALP staining kit (Cell Biolabs, San Diego, CA, USA) was used to measure the ALP activity on day 4 and 7 post treatment (Fig. 8A).

ARS staining was performed to quantify the mineralized bone nodules, as another marker for osteoblastic activity. Briefly, cells were treated with teriparatide for 28 days and because of the overgrowth of Mtb and MG-63 cells, the cell numbers and MOI were reduced to 5 × ${10}^{3}$ cells/well and 0.1 MOI, respectively (Fig. 8B). The number of red nodules observed with ARS staining was counted under a microscope. The stained wells were quantified using a microscope interfaced with a digital camera and a bioquantification software system (Bioquant Osteo II, R&M Biometrics, Nashville, TN, USA) using a previously described method^67^. We confirmed the effect of teriparatide treatment on bone volume/tissue volume ratio using a bioquantification analysis software. Bone volume was defined as the ALP- and ARS (bone nodule area)-positive areas in the ALP and ARS staining assays, respectively.

### Elisa

Culture supernatants were harvested at each time point shown in Fig. 7, passed through a 0.2 $\upmu$m spin filter to remove viable organisms, and frozen before assaying for chemokine proteins. The levels of interleukin (IL-8), monocyte chemoattractant protein-1 (MCP-1), interferon γ-induced protein 10 kDa (IP-10), and regulated upon activation normal, T cell expressed and presumably secreted (RANTES) were detected using ELISA with matched pairs of antibodies (R&D Systems, Minneapolis, MN, USA) according to the manufacturer’s instructions. The lower limit of sensitivity of the IP-10 assay was 11 pg/mL and that of all other ELISAs was 3 pg/mL. The results are expressed as picogram per milliliters (pg/mL) and are presented as means ± standard error of the mean (SEM) of at least three experiments.

## Data Availability

Upon request by e-mail to the corresponding author, we (including all co-authors) will confirm the use and provide research materials.
